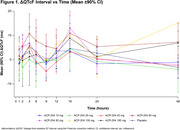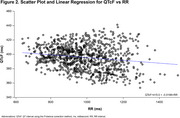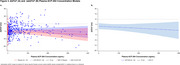# Analyzing the Effect of ACP‐204 on the QTc Interval in Healthy Adult Participants Using Phase 1 Study Data

**DOI:** 10.1002/alz70859_105893

**Published:** 2025-12-25

**Authors:** Mona Darwish, Jay W Mason, Stephanie W Stanworth, Xiaoshu Feng, Bryan Dirks, Brian Raether, Sanjeev S Pathak

**Affiliations:** ^1^ Acadia Pharmaceuticals Inc., Princeton, NJ USA; ^2^ Mason Cardiac Safety Consulting, Reno, NV USA

## Abstract

**Background:**

QT interval prolongation is a known adverse event of many psychotropic medications. ACP‐204, a potent and selective inverse agonist/antagonist of serotonin 2A (5‐HT_2A_), was developed for the treatment of Alzheimer’s disease psychosis to have an improved pharmacological profile and lower risk of QT prolongation. This study evaluates the effect of ACP‐204 on corrected QT (QTc) intervals and the relationship between plasma drug concentrations and time‐matched change in QTc following single doses in healthy volunteers.

**Method:**

QTc intervals were analyzed from randomized, placebo‐controlled, double‐blind, Phase 1 data in which healthy adult participants were randomized to single ascending oral doses of ACP‐204 (10 to 180 mg) or placebo. Data from 12‐lead electrocardiogram (ECG) assessments at screening, baseline, and up to 48 hours postdose were used to evaluate changes from baseline in Friderica‐corrected QT (ΔQTcF) intervals. Concentration‐effect modeling was used to analyze the relationship between plasma drug concentrations and time‐matched changes in QTcF. QTc interval assessments were summarized descriptively and categorically.

**Result:**

This analysis included 57 participants. A ΔQTcF >30 ms and ≤60 ms was observed in 1 participant in each of the 60, 130, and 180 mg cohorts; 1 participant in the 40 mg cohort had a ΔQTcF >60 ms. The QTcF interval did not exceed 450 ms at any time point for any participant, and no dose‐response pattern was observed (Figure 1). The Fridericia heart rate correction met adequacy criterion; 89.5% of participants had QTcF versus RR interval slopes <|0.045|, exceeding the 50% requirement (Figure 2). Changes observed using concentration‐effect modeling were small and benign (Figure 3A‐B). After subtraction of placebo ΔQTcF, the upper limit of the confidence band remained below 10 ms throughout to the maximum observed concentration of ∼395 ng/mL. Model‐predicted average placebo‐adjusted ΔQTcF (ΔΔQTcF) at mean C_max_ levels for all dose cohorts ranged from 3.17 ms (10 mg) to 0.47 ms (180 mg). The upper limit of the 2‐sided 90% CI for ΔΔQTcF at 180 mg was 6.51 ms.

**Conclusion:**

No meaningful increases in QTcF were observed in healthy participants after single‐dose administration of ACP‐204 (10‐180 mg); specifically, there was no QTcF prolongation up to 180 mg.